# Using routine inpatient data to identify patients at risk of hospital readmission

**DOI:** 10.1186/1472-6963-9-96

**Published:** 2009-06-09

**Authors:** Stuart Howell, Michael Coory, Jennifer Martin, Stephen Duckett

**Affiliations:** 1 Centre for Healthcare Improvement, Queensland Health, Brisbane, Australia; 2 School of Population Health, University of Queensland, Brisbane, Australia; 3 Department of Internal Medicine, Royal Brisbane and Women's Hospital, Brisbane, Australia; 4 Diamantina Institute, University of Queensland, Brisbane, Australia

## Abstract

**Background:**

A relatively small percentage of patients with chronic medical conditions account for a much larger percentage of inpatient costs. There is some evidence that case-management can improve health and quality-of-life and reduce the number of times these patients are readmitted. To assess whether a statistical algorithm, based on routine inpatient data, can be used to identify patients at risk of readmission and who would therefore benefit from case-management.

**Methods:**

Queensland database study of public-hospital patients, who had at least one emergency admission for a chronic medical condition (e.g., congestive heart failure, chronic obstructive pulmonary disease, diabetes or dementia) during 2005/2006. Multivariate logistic regression was used to develop an algorithm to predict readmission within 12 months. The performance of the algorithm was tested against recorded readmissions using sensitivity, specificity, and Likelihood Ratios (positive and negative).

**Results:**

Several factors were identified that predicted readmission (i.e., age, co-morbidities, economic disadvantage, number of previous admissions). The discriminatory power of the model was modest as determined by area under the receiver operating characteristic (ROC) curve (c = 0.65). At a risk score threshold of 50, the algorithm identified only 44.7% (95% CI: 42.5%, 46.9%) of patients admitted with a reference condition who had an admission in the next 12 months; 37.5% (95% CI: 35.0%, 40.0%) of patients were flagged incorrectly (they did not have a subsequent admission).

**Conclusion:**

A statistical algorithm based on Queensland hospital inpatient data, performed only moderately in identifying patients at risk of readmission. The main problem is that there are too many false negatives, which means that many patients who might benefit would not be offered case-management.

## Background

Containing the cost of acute hospital services has been a feature of health systems around the world since at least the 1980s [1]. Strategies include increased use of day surgery, reduction in the number of long-stay beds, reduction in length of stay for overnight patients, and increased acute care at home (hospital in the home).

Another strategy for containing costs is to improve case-management of patients who are at high risk of re-admission. This is attractive because a small percentage of patients account for a much larger percentage of inpatient costs. For example, a study in Western Australia reported that the top 5% of hospital users accounted for 38% of inpatient costs and 26% of inpatient separations [2]. This top 5% mainly comprised patients with chronic medical conditions.

There is some evidence that case-management of patients with chronic medical conditions reduces readmissions and improves health outcomes and quality of life [3-10]. The precise elements vary depending on the setting, but typically it comprises nurse-centred discharge planning and post-discharge support (e.g., home visits or telephone calls) with the aim of improving co-ordination of specialist and primary health care, optimising therapy, and ensuring timely access to the available medical and social services.

Assuming that an effective case-management intervention can be developed for a particular setting, it would be advantageous to identify patients at high risk of readmission in advance so that case-management can be targeted to those who would benefit most. This would reduce the overall costs of the intervention and maximise the benefits. In the United Kingdom (UK) [11-13] and the United States (US) [14], case-finding algorithms for patients at risk of readmission have been developed using administrative and patient data. The aim of this study was to develop a predictive algorithm using Australian (Queensland) routine inpatient data and to establish whether this would provide an effective method of identifying patients at risk of hospital readmission. We evaluated the algorithm on the basis of sensitivity, specificity and predictive values.

## Methods

### Data

Data for public-sector patients were obtained from the Queensland Hospital Admitted Patients Data Collection (QHAPDC), which contains, *inter alia*, the demographic characteristics of the patients, the principal diagnosis, other conditions treated, and the procedures performed. QHAPDC is similar to routine inpatient databases in the other states and territories of Australia and is unlikely to differ substantially from those in the UK.

### Inclusion criteria

We used a list of 28 *reference *conditions (e.g., congestive heart failure, chronic obstructive pulmonary disease, diabetes, dementia) that was as close as possible to those used in the UK study within the constraints of Australian coding standards (Table 1) [15]. (The US study included adult disabled patients who were eligible for mandatory enrolment in Medicaid managed care [14]. This subgroup could not be replicated with Australian data.)

**Table 1:** Reference conditions used to define cases for the predictive algorithm

AR DRG codes*	Description
B63Z	Dementia and other chronic disturbances of cerebral function
B67A-B67C	
B68A-B68B	Multiple sclerosis and other demyelinating disorders
B76A-B76B	Epilepsy (<69 years or with complications or co-morbidities)
E60A-E60B	Cystic fibrosis
E65A-E65B	Chronic obstructive pulmonary disease (includes bronchiectasis)
E69A-E69B	Asthma (>49 years or with complications or co-morbidities)
E74A-E74C	Interstitial lung disease
E75A-E75B	Other respiratory diagnoses (>69 years or with complications or co-morbidities)
E60A-E75B; (excluding E71A-E71C, lung cancer)	Complex elderly with a respiratory system primary diagnosis (>69 years)
F62A-F62B	Heart failure
F65A-F65B	Peripheral vascular disorders (>69 years or with complications or co-morbidities)
F66A-F66B	Coronary atherosclerosis
F70A-F71B	Arrhythmia or conduction disorders (>69 years or with complications or co-morbidities)
F72A-F72B	Angina (>69 years or with complications or co-morbidities)
F60A-F75C	Complex elderly with a cardiac primary diagnosis (>69 years)
	
I65A-I70Z	Inflammatory spine, joint or connective tissue disease
J60A	Skin ulcers
K60A-K60B	Diabetes (>69 years or with complications or co-morbidities)
K01Z	Diabetic foot procedures
K61Z-K64B	Complex elderly with endocrine or metabolic system primary diagnosis (>69 years)
L63A-L63B	Kidney or urinary tract infections (>69 years or with complications or co-morbidities)
Q61A-Q61C	Red blood cell disorders (>69 years or with complications or co-morbidities)
Q62Z	Coagulation disorders

* Australian Refined Diagnosis Related Groups [15]

The UK reference list is intended to include those conditions for which timely and effective case-management has the potential to reduce the risk of readmission. As discussed in the UK and US papers, a large percentage of hospitalisations cannot be prevented even with the most effective case-management or are episodic with repeated admissions in one year, but not in subsequent years; examples include major trauma and cancer [11,14]. For these and other similar conditions, the need for hospitalisation is largely driven by factors beyond the control of an outpatient, case-management intervention, at least in the medium or short term.

Patients were selected for inclusion in the study if they had an emergency inpatient admission for a reference condition during the financial year 2005/2006. These admissions represented 15% of all emergency medical inpatient admissions during the period. Medical inpatient admissions were identified using AR-DRG codes [15] and emergency inpatient admissions were defined as those, which, in the opinion of the treating physician, could not be delayed for more than 24 hours [16].

If the patient had more than one emergency medical admission during 2005/2006, we took the first admission as the triggering admission for consistency with earlier studies. Sensitivity analyses (not presented) showed that, for patients who had more than one admission in 2005/2006, it made no difference to the results whether we used the first or the last admission during 2005/2006 as the triggering admission. Patients who died during the triggering admission were removed from the analysis.

### Outcome measure

We classified a patient as being readmitted if, within the 12 months following discharge for the triggering admission, they had at least one acute admission. We excluded planned, same-day admissions from this definition because in Queensland public hospitals such admissions are predominantly for regular and recurring treatments (dialysis, chemotherapy) or for diagnostic procedures (endoscopy), which are often not included as inpatient episodes-of-care in other health systems.

### Predictor variables

#### Demographic measures

Demographic characteristics were obtained from the trigger admission and included age, sex, Indigenous status, marital status, socioeconomic status and rurality (as a potential marker of access to hospital care). Indigenous status is routinely collected in the QHAPDC and was classified as Indigenous versus Non-Indigenous. Socioeconomic status was characterised using the SEIFA (Socio Economic Indexes For Areas) Index of Advantage/Disadvantage (Australian Bureau of Statistics (ABS), 2004) [17]. This is a composite measure, which describes area advantage/disadvantage according to a range of social and economic factors as determined from census data. Geographic remoteness was characterised using the Accessibility/Remoteness Index of Australia (ARIA: ABS, 2004) [18]. This measures the remoteness of a physical location based on road distance to the nearest urban centre(s). SEIFA and ARIA index values were mapped onto QHAPDC data using Statistical Local Area of usual residence. A quintile split of SEIFA values was used to create five socioeconomic groups ranging from most disadvantaged (Quintile 1) to most advantaged (Quintile 5). Area remoteness was summarized using five major categories in accordance with ABS cut points: Major city (score: 0–0.20), Inner Regional (>0.20–2.40), Outer Regional (>2.40–5.92), Remote (>5.92–10.53) and Very Remote (>10.53).

#### Co-morbidities

The presence of co-morbidities was identified from the primary and from all nine secondary diagnosis fields for the trigger admission and all acute admissions in the 3 years before the triggering event (Table 2). The decision to evaluate comorbidities across all admissions up to and including the trigger admission was in order to account for potential lapses in diagnostic coding. The comorbid conditions evaluated in this study were similar to those reported in the UK study as listed on the King's Fund website [19].

**Table 2:** Co-morbid conditions evaluated in the development of the risk algorithm

ICD10-AM	Co-morbidity	ICD10-AM	Co-morbidity
D5000–D6499	Anaemia	I2000–I2599	Acute coronary syndrome

I4800–I4899	Cardiac arrhythmias	I5000–I5099	Heart failure

I1000–I1599	Hypertensive diseases	I6000–I6999	Cerebrovascular diseases

I7000–I7399	Peripheral vascular disease	C0000–C9699	Malignant neoplasms

J4500–J4699	Asthma	J4700–J4799	Bronchiectasis

J4000–J4499	COPD including bronchitis and emphysema	G2000–G2299	Parkinson's disease

G3000–G3099 F0000–F0399	Dementias	F1000–F1099	Mental disorders due to alcohol use

F1100–F1999	Mental disorders due to other drug use	E1000–E1499	Diabetes mellitus

N1700–N1799	Acute renal failure	N1800–N1999	Chronic renal failure

J1000–J2299	Lower respiratory tract infections	M0500–M1499	Inflammatory arthritis

M3000–M3699	Systemic connective tissue disorder	F2000–F2999	Schizophrenic disorders

F3000–F3999	Mood (affective) disorders	I0500–I0999 I3300–I3999	Chronic rheumatic heart disease and other heart valve disorders

K7000–K7799	Liver diseases	K8500–K8699	Pancreatic diseases

B2000–B2499	HIV	M8000–M8299	Osteoporosis

W0000–W1999	Falls		

#### Prior utilisation

For consistency with the UK study, previous admissions were enumerated for three time intervals preceding the trigger admission – 90 days, one year and three years – for two admission categories: any admission and any emergency admission. The measures displayed the intrinsic skewness of hospital utilisation data, which was resolved by treating each measure as an ordinal variable classified as 0, 1, 2 or more admissions. As with the outcome measure, we excluded planned, same-day admissions from this definition, and we also excluded sub-acute and non-acute admissions (e.g.: admissions for rehabilitation or palliative care).

### Statistical analysis

#### Predictive algorithm

The predictive algorithm was developed using logistic regression as implemented by SAS Version 9.1. The predictor measures were first evaluated within their natural classes to minimise co-linearity and to prevent a large number of conceptually similar measures from saturating the model. For example, the socio-demographic variables were entered into a regression model as a group and the best subset of them was identified using the purposeful selection methods proposed by Hosmer and Lemeshow [20]. The *best *subsets of co-morbidities and of utilisation measures were identified in a similar way. These subsets were then combined and further backward elimination was applied to identify the most parsimonious model. The Likelihood Ratio was assessed to evaluate successive models. Variables were considered as candidates for the model if they were univariately significant at alpha < 0.25. We retained variables that remained significant at alpha < 0.10, as well as those that were identified as confounders [20]. Significant categorical and nominal variables were retained in complete form – that is, all levels of the variable were retained in the model.

The predictive algorithm was developed using a 75% training sample selected at random of *triggering *admissions and validated on the remaining 25%. This cut point was chosen in recognition of the small sample relative to those in other studies, and the large number of candidate variables being evaluated in developing the algorithm. Results from the sensitivity and specificity analysis were similar in the two samples (i.e. varied by < 3%), and findings from the validation sample are reported here.

#### Performance of algorithm

The regression coefficients were applied to the validation sample and a predicted probability of readmission was generated for each individual from the coefficients. For consistency with the UK [11] and US studies [14], we assessed the performance of the algorithm using sensitivity (percentage of patients readmitted in the next 12 months who were correctly identified by the algorithm); specificity (percentage of patients not readmitted in the next 12 months who were not flagged by the algorithm); and the false positive rate (percentage of patients flagged by the algorithm who were not be admitted in the next 12 months). Sensitivity, specificity and the false positive rate were estimated by comparing the actual readmissions with readmissions as predicted by the algorithm; these were evaluated using three separate cut-points of predicted risk from the algorithm (i.e., the logistic regression model): 50%, 70% and 80%.

We also calculated the likelihood ratio for being readmitted (LR+) and the likelihood ratio for not being readmitted (LR-).

, and is the increase in odds of having a readmission, given that the algorithm has flagged.

, and is the decrease in odds of having a readmission, given that the algorithm has not flagged.

There are established benchmarks for likelihood ratios when they are used to assess the performance of diagnostic tests and we used these benchmarks in this study [21]. The predictive ability of the algorithm was characterised using the LR+ as follows: Excellent (LR+ greater than 10.0), Good (6.0 < = LR+ < = 10.0), Fair (2.0 < = LR+ < = 5.9) and Poor (1.0 < = LR+ < = 1.9); the corresponding ranges for the LR- were: Excellent (LR- < 0.1), Good (0.1 < = LR- < = 0.2), Fair (0.3 < = LR- < = 0.5) and Poor (0.6 < = LR- < = 1.0). The area under the receiver operating curve (ROC) was also evaluated to determine the predictive ability of the algorithm.

Queensland Health, the data custodian, advised that research ethics approval was not required for this study because the analyses did not use any data that could identify (or potentially identify) an individual patient and because all analyses were completed on Queensland Health premises by a Queensland Health employee (SH). The analyses were done in a secure computing environment in a physically locked area. Access to the computer system is password protected, is subject to monitoring through audit trails and is only accessible to authorised staff.

## Results

Table 3 shows the demographic characteristics for the full sample and separately for the development and validation samples. During the financial year 2005/2006, 17,699 people had at least one emergency admission for a reference condition to a Queensland public hospital. The male to female ratio was 49% versus 51%. Age ranged from 0 to 104 years with a mean of 66.0 years and was similar among females and males (mean age: 67.6 versus 64.4 years). Only 3% were younger than 18 years, in contrast to emergency admissions for patients without a reference condition, where 20% were younger than 18 years. The 12-month readmission rate for patients with at least one admission for a reference condition was 45% (95% CI: 44.7% – 46.1%). There were no obvious differences between the development and validation samples, as would be expected if subjects were assigned at random.

**Table 3:** Characteristics of the full patient sample and for the Development and Validation samples

	Full Sample (n = 17,699)	Development sample (n = 13,207)	Validation sample (n = 4,492)
	%	%	%

Twelve month readmission rate	45.4	45.5	45.1

Age Group			
0–17 years	3.2	3.2	3.3
18–39 years	8.8	8.7	9.0
40–64 years	23.7	23.8	23.4
65–74 years	20.5	20.4	20.7
75+ years	43.8	43.9	43.6

Sex			
Male	49.3	49.4	48.9
Female	50.7	50.6	51.1

Marital status			
Never married	20.6	20.5	20.8
Married/de facto	48.6	48.8	48.1
Widowed	21.0	20.9	21.2
Separated/divorced	9.9	9.8	9.9

Indigenous status			
Non-Indigenous	96.0	95.9	96.1
Indigenous	4.0	4.1	3.9

SEIFA^1^			
Most disadvantaged	28.5	28.8	27.8
Quintile 2	28.0	27.8	28.5
Quintile 3	21.0	21.1	20.7
Quintile 4	15.7	15.6	16.2
Least Disadvantaged	6.7	6.7	6.8

ARIA^2^			
Major city	43.7	43.3	44.8
Inner Regional	28.6	28.9	27.9
Outer Regional	22.6	22.6	22.6
Remote	2.7	2.8	2.4
Very Remote	2.4	2.5	2.4

^1^Socio-Economic Indexes for Areas

^2^Accessibility/Remoteness Index of Australia

### Predictors included in the algorithm

Regression parameters for the predictive algorithm are presented in Table 4. The final model included 16 of the 41 predictors that were evaluated. Readmission was associated with a broad range of demographic measures, co-morbidities and hospital utilisation measures, although the odds ratios were modest. For prior utilisation, *'any previous admission' *was highly correlated with '*previous emergency admission' *and both could not be included in the algorithm because of co-linearity. Table 4 shows the odds ratios for any previous admission; the odds ratios and performance of the algorithm were similar when '*previous emergency admission*' was used. We retained the model with *'any previous admission' *on the grounds that '*previous emergency admission*' was a subset of the former category and it did not provide superior explanatory power.

**Table 4:** Odds ratios for the final variables retained in the predictive algorithm

Parameter	Odds ratio	95% CI	p-value
Age Group			
0–17 years	1.00	-	
18–39 years	0.94	0.74–1.19	0.60
40–64 years	1.09	0.88–1.36	0.43
65–74 years	1.32	1.06–1.65	0.02
75+ years	1.30	1.05–1.61	0.02

SEIFA^1^			
Most disadvantaged	1.00	-	
Quintile 2	0.85	0.78–0.94	0.001
Quintile 3	0.96	0.86–1.06	0.39
Quintile 4	0.79	0.71–0.89	0.0001
Least Disadvantaged	0.80	0.68–0.94	0.007

ARIA^2^			
Major city	1.00	-	
Inner Regional	1.01	0.92–1.11	0.81
Outer Regional	1.10	1.00–1.22	0.04
Remote	1.30	1.04–1.62	0.02
Very Remote	1.36	1.08–1.73	0.01

Co-morbidities			
Anaemia	1.31	1.16–1.47	0.001
Acute coronary syndrome	1.14	1.04–1.25	0.005
Heart failure	1.25	1.12–1.38	<0.0001
Peripheral vascular disease	1.32	1.10–1.58	0.003
Malignant neoplasms	1.29	1.10–1.51	0.002
COPD	1.21	1.10–1.34	0.0002
Mental disorder (alcohol)	1.42	1.16–1.74	0.001
Diabetes	1.27	1.16–1.39	<0.0001
Chronic renal failure	1.30	1.12–1.52	0.001
Mood disorders	1.22	1.00–1.50	0.06
Liver diseases	1.45	1.06–1.99	0.02

Any previous admission			
Past year			
None	1.00	-	
One	1.14	1.01–1.28	0.03
Two or more	1.63	1.38–1.91	<0.0001
Past 3 years			
None	1.00	-	
One	1.45	1.31–1.62	<0.0001
Two or more	1.74	1.53–1.98	<0.0001

^1^Socio-Economic Indexes for Areas

^2^Accessibility/Remoteness Index of Australia

The predictive algorithm had a sensitivity of 44.7% at a risk score threshold of 50% (Table 5). That is, the algorithm identified less than one-half of the patients who were readmitted over the following twelve months. The false positive rate was 37.5%, which indicates that more than one-third of the patients who were identified at being at risk of readmission were incorrectly flagged. The false positive rates (and the specificity) improved at the higher risk thresholds of 70% and 80% (28.2% and 14.8%, respectively), however, the model's ability to identify readmitted patients was substantially compromised as indicated by sensitivities of 7.8% and 1.1%, respectively. The receiver operating characteristic (ROC) score was 0.65, indicating modest model discrimination. The LR+ was fair at the 50% and 70% risk-score thresholds (Table 5: 2.04 and 3.11) and was good at the 80% risk-score thresholds (7.02); the LR- was poor at all risk-score thresholds (Table 5: 0.7–1.0).

**Table 5:** Performance of the algorithm in predicting readmission for Queensland compared to UK and US data

	Risk score threshold		
	50%	70%	80%

**Queensland data**			
Sensitivity (95% CI)	44.7 (42.5–46.9)	7.8 (6.7–9.1)	1.1 (0.1–1.7)
Specificity (95% CI)	78.1 (76.4–79.6)	97.5 (96.8–98.1)	99.8 (99.6–99.9)
False positive rate (95% CI)	37.5 (35.0–40.0)	28.2 (22.3–34.6)	14.8 (4.2–33.7)
LR+ (95% CI)	2.04 (1.86–2.23)	3.11 (2.33–4.15)	7.02 (2.43–20.28)
LR- (95% CI)	0.71 (0.68–0.74)	0.95 (0.93–0.96)	0.99 (0.99–1.00)

**UK study**[11]			
Sensitivity	54.3	17.8	8.1
Specificity	72.2	95.0	98.6
False positive rate	34.7	22.6	15.7
LR+ (95% CI)	2.0	3.6	5.8
LR- (95% CI)	0.6	0.9	0.9

**US study**[14]			
Sensitivity	57.9	Not reported	Not reported
Specificity	70.7		
False positive rate	34.1		
LR+ (95% CI)	2.0		
LR- (95% CI)	0.6		

## Discussion

This study shows that routine-inpatient data for public-hospital patients in Queensland can be used to develop a case-finding algorithm with statistical characteristics that are similar to those for algorithms developed using UK [11] and US data [14]. The false positive rate (37.5%) for this study (at a risk-score threshold of 50%) was similar to, although slightly higher than, that reported in the UK (34.7%) and US (34.1%). The sensitivity for this study was 44.7% (at a risk-score threshold of 50%), which is similar to, although slightly lower than, the corresponding percentages obtained from the UK (54.3%) and US (57.9%) studies. The ROC score for this study was modest (0.65), and similar to that for the UK (0.69) study. (We could not locate a published ROC score for the US study.)

Likelihood ratios are a convenient way of summarising the performance of a predictive algorithm (Table 3). LR+ for the Australian and UK and US algorithms were generally fair, but all the LR- were poor and for higher risk-score thresholds they were close to 1.0. This is because algorithms based on routine inpatient data have poor sensitivity (i.e., large percentage of false negatives). To give this some context, LR- associated with carcinoembryonic antigen (CEA) for detecting early colorectal cancer are also close to 1.0, so that after some initial enthusiasm [22], it was decided that CEA should not used as screening test for colorectal cancer.

This is not to say that the risk of readmission cannot be assessed with any confidence; merely that the strongest predictors might be difficult to measure in a reproducible way. For example, critical factors might be whether the patient's general practitioner is comfortable in dealing with the current severity of the patient's condition or whether the patient's family are available, at this particular time, to assist. Also, there might be a constellation of circumstances (all difficult to measure) that might mean that readmitting the patient to hospital is easier and/or safer than organising the required tests and services (including pharmacy and meals-on-wheels) as an outpatient.

### Potential limitations of this study

This study has potential limitations which may have influenced the statistical performance of the algorithm. First, the age range was wide (0–104 years), and this may have diluted the power of the algorithm as factors are likely to differ between children and the elderly. Further, the algorithm may have performed better in a more homogenous patient group. This is some evidence in support of both points. For instance, Donnan and associates [13] have recently developed a predictive algorithm amongst patients aged 40 years and older, which provided better discriminatory power (as assessed by area under the receiver operator curve (c = 0.8) than algorithms reported in our study and elsewhere [11,14]. Similarly, the US study [14] reported an algorithm developed amongst patients with serious and persistent mental illness, and obtained a sensitivity at the risk-score of 50% of 77%, which is getting close to values needed for a useful algorithm.

It is also possible that the algorithm may have performed better if we had applied a stricter definition to our outcome measure. An algorithm which predicts readmission over a shorter time frame – for example, three months – may perform better than one which predicts the risk of readmission over the following year, although this would require further investigation. Similarly, it may be preferable to predict the risk of becoming a "frequent" user of hospital care, as defined by the number of readmissions over the following year. There is limited evidence to support this possibility. For example, Bottle et al [12] have recently reported the results of three algorithms that were developed to predict the 12-month risk of two more admissions in a sample of patients following an emergency admission. All three models showed better discrimination than our algorithm, although the performance was only a modest improvement over the earlier UK model.

Unlike the UK and US studies [11,14], we did not have information on non-admitted care and this might explain why the Queensland algorithm performed slightly worse than UK and US algorithms. However, the differences in performance were small (Table 5), suggesting that data-items for non-admitted care are not strong predictors of readmission.

### Cost

The UK and US studies included business cases [11,14] to show that case-management would be cost-saving if applied to the small number of patients for whom the algorithm predicted that the risk of readmission was > 70% (i.e., case-management would cost less than the cost of the readmissions it prevented). Only a very few interventions (e.g., childhood immunisation) have been identified that both improve health outcomes and are cost-saving, so it would be unusual if case-management proved to be cost-saving. Much more commonly, we have to spend money to improve health and quality of life, although we would want to give priority to interventions that are cost-effective (value-for-money). Given the high cost of readmission and the high average risk of readmission for patients with a reference condition (45% for these Queensland data), it is likely that a case-management intervention offered to all patients with a reference condition could be value-for-money, but perhaps not cost-saving.

## Conclusion

A statistical algorithm, uninformed by clinical judgement, is unlikely ever to be an appropriate way to identify patients in need of care or additional interventions. Perhaps the most appropriate use of a statistical algorithm is to identify those patients who might benefit from closer clinical attention. The algorithm could thus be used to highlight patients presenting to the emergency department (on a second visit), to assist emergency physicians to identify quickly those patients at higher risk of readmission for potential referral to case management. With intervening clinical judgement, false positives should be reduced, but unless the risk thresholds are reduced further (at the cost of increased emergency physician time in reviewing cases), false negatives would remain an issue. This then becomes a policy question of whether a benefit to one group (those identified) should be introduced even though others who might benefit have been (unfairly) not so identified, with their access to additional interventions relying on (unprompted) clinical judgement.

## Competing interests

The authors declare that they have no competing interests.

## Authors' contributions

SH extracted and analysed the data and prepared a draft of the manuscript. MC prepared a revised version of the manuscript and provided intellectual input into study design and statistical analysis. JM and SD provided content expertise throughout the design and implementation of the study and contributed to the final version of the manuscript.

## Pre-publication history

The pre-publication history for this paper can be accessed here:


